# Heterogeneous impact of tea consumption on COPD risk in smokers: insights from the PIFCOPD study

**DOI:** 10.3389/fmed.2026.1776347

**Published:** 2026-03-18

**Authors:** Yijing Li, Jiping Liao, Ruiying Wang, Xiuhua Fu, Xiaomin Dang, Hua Qiao, Lixia Dong, Jianhong Xiao, Shujuan Jiang, Jinzhi Yin, Weihua Jia, Xixin Yan, Yunxia Wang, Cheng Zhang, Kunyao Yu, Guifang Zhang, Jing Li, Rui Chen, Bo Zhou, Mengyu Yin, Shaochen Dong, Jian Sun, Peng Gao, Bifang Miao, Beibei Song, Liping Xie, Lan He, Qian Ning, Lina Zhang, Wei Li, Qi Zhang, Kunyan Sun, Chunbo Zhang, Xiaoyu Ma, Guangfa Wang

**Affiliations:** 1 Department of Respiratory and Critical Care Medicine, Peking University First Hospital, Beijing, China; 2 Department of Pulmonary and Critical Care Medicine, Shanxi Bethune Hospital, Shanxi Academy of Medical Sciences, Tongji Shanxi Hospital, Third Hospital of Shanxi Medical University, Taiyuan, Shanxi, China; 3 Division of Pulmonary and Critical Care Medicine, The Affiliated Hospital of Inner Mongolia Medical University, Hohhot, Inner Mongolia, China; 4 Department of Respiratory and Critical Care Medicine, Xi'an Jiaotong University Medical College First Affiliated Hospital, Xi'an, Shaanxi, China; 5 Department of Respiratory and Critical Care Medicine, The First Hospital of Qinhuangdao, Qinhuangdao, Hebei, China; 6 Department of Respiratory and Critical Care Medicine, Tianjin Medical University General Hospital, Tianjin, China; 7 Department of Respiratory and Critical Care Medicine, Mindong Hospital of Ningde City, Ningde, Fujian, China; 8 Department of Respiratory and Critical Care Medicine, Shandong Provincial Hospital Affiliated to Shandong First Medical University, Jinan, Shandong, China; 9 Department of Respiratory and Critical Care Medicine, The Second Hospital of Jilin University, Changchun, Jilin, China; 10 General Hospital of Taiyuan Iron & Steel (Group) Co., LTD., Taiyuan, Shanxi, China; 11 Department of Respiratory and Critical Care Medicine, Hebei Key Laboratory of Respiratory Critical Care, The Second Hospital of Hebei Medical University, Shijiazhuang, Hebei, China; 12 Jinyuan Community Health Service Centre, Taiyuan, Shanxi, China; 13 Jining First People's Hospital, Jining, Shandong, China

**Keywords:** chronic obstructive pulmonary disease, interaction effect, polyphenols, smoking, tea consumption

## Abstract

**Background:**

Chronic obstructive pulmonary disease (COPD) is a highly prevalent disease worldwide, with smoking identified as the main risk factor. Emerging evidence suggests that tea consumption may confer protective effects on lung health. However, it remains unclear whether tea consumption can mitigate the adverse effects of smoking on COPD risk. This study aimed to investigate the association between tea consumption and COPD risk, and to examine whether this association differs by smoking status.

**Methods:**

The Predictive Value of Inflammatory Biomarkers and Forced Expiratory Volume in 1 s (PIFCOPD) study is a multicenter prospective cohort (2018–2021) involving 7,252 participants (6,855 with normal lung function; 397 with COPD). Data on demographic characteristics, smoking status, and tea consumption were collected. Logistic regression analysis was used to assess the association between tea consumption and COPD risk. Interaction and stratified analyses based on smoking status were performed.

**Results:**

The proportion of tea consumption was 20.9% in the normal lung function group and 24.4% in the COPD group, with no significant difference. Tea consumption was not associated with COPD risk in the general population. However, interaction and stratified analysis based on smoking status showed that fully fermented tea consumption was associated with a protective effect against COPD in smokers (OR 0.21), especially when consumed ≥ 7 times per week and for ≥ 10 years. In contrast, jasmine tea emerged as a potential risk factor (OR 1.99), especially when consumed for ≥ 10 years.

**Conclusion:**

In the general population, the effect of tea consumption on the prevalence risk of COPD is significant only in smokers, and this effect is modulated by the type, frequency and duration of tea consumption.

## 1 Introduction

Chronic Obstructive Pulmonary Disease (COPD) is a progressive respiratory disease characterized by persistent airflow limitation and chronic airway inflammation (1). With an estimated global prevalence of 10.3%, it is the third leading cause of death worldwide and contributes significantly to the global disease burden (1, 2). The pathogenesis of COPD involves a multifactorial interplay between environmental exposures and genetic susceptibility (3). Among these, long-term inhalation of harmful gases and particulate matter-most notably tobacco smoke-remains the most critical risk factor (1, 3). Cigarette smoke exposure induces excessive production of reactive oxygen species (ROS), leading to sustained oxidative stress, activation of pro-inflammatory signaling pathways, and impairment of endogenous antioxidant defenses (4). These processes promote chronic airway inflammation, protease-antiprotease imbalance, tissue remodeling, and progressive decline in lung function, which together constitute the core pathophysiological features of COPD (5, 6). The key to the management of COPD is early intervention, as most patients are diagnosed at moderate to advanced stages (7). The primary strategies for early prevention are smoking cessation and pollutant reduction, while evidence supporting other lifestyle-based interventions remains inconclusive.

Given the central role of chronic inflammation and oxidative stress in COPD pathophysiology, increasing attention has been directed toward the potential modifying effects of diet on respiratory health (8). Diets rich in antioxidants and anti-inflammatory components have been associated with lower systemic inflammation and reduced oxidative injury, suggesting a potential role in influencing the risk and progression of chronic respiratory diseases, including COPD (9). Among dietary bioactive compounds, phytochemicals-particularly polyphenols-have demonstrated antioxidant and anti-inflammatory properties, providing a biologically plausible basis for investigating polyphenol-rich foods and beverages in relation to smoking-related airway injury (10).

Tea, derived from the leaves of *Camellia sinensis*, is the second most popular beverage worldwide, after water. Over 60% of global tea consumption occurs in Asia, with China, India, and Japan being the leading consumers (11). Based on the degree of fermentation, tea is commonly categorized into four main types: unfermented (such as green tea), partially fermented (such as oolong tea), fully fermented (such as black tea), and post-fermented tea (such as dark tea) (12). Additionally, reprocessed teas, such as jasmine tea, are also commonly consumed. Globally, black tea accounts for approximately 78% of total consumption, green tea for 20%, and other types for less than 2% (13).

Tea contains abundant polyphenolic compounds, which have been reported to attenuate cigarette smoke-induced lung injury through modulation of inflammatory and oxidative stress-related mechanisms (10, 14). Epidemiologic evidence on tea consumption and COPD has also emerged. A Korean population-based study reported that consuming green tea ≥2 times/day was associated with a lower prevalence of COPD, while a cohort study in Singapore evaluated different tea types and suggested a potential protective association in older adults (15, 16). However, existing evidence remains limited and inconsistent. Prior studies have primarily focused on selected populations and specific tea types, with limited characterization of drinking patterns (type, frequency, and duration) and insufficient evaluation of effect modification by smoking.

As China is a major consumer of tea, understanding the effects of tea on COPD especially in smokers, is important for public health. This study examines the potential protective effects of tea consumption on COPD using data from a large community, focusing on high-risk groups such as smokers, to inform prevention strategies.

## 2 Materials and methods

### 2.1 Study design and participants

The Predictive Value of Inflammatory Biomarkers and Forced Expiratory Volume in 1 s (FEV_1_) for Chronic Obstructive Pulmonary Disease (PIFCOPD) study is a nationwide, multicenter, prospective cohort study conducted between 2018 and 2021. The participating centers were located in Beijing, Fujian, Hebei, Henan, Inner Mongolia, Jilin, Shandong, Shanxi, Tianjin, and Shaanxi. The cohort enrolled community-dwelling adults aged 40–75 years. Individuals with interstitial lung disease, a history of lung surgery, or other conditions known to cause abnormal pulmonary function were excluded. Detailed inclusion and exclusion criteria of the original cohort have been reported previously (17). For the present analysis, participants with unacceptable-quality spirometry were excluded. Normal lung function was defined as a FEV_1_/forced expiratory volume in 6 s (FEV_6_) ratio ≥ 70% and an FEV_1_ ≥ 80% of the predicted value, whereas COPD was defined as a post-bronchodilator FEV_1_/FEV_6_ ratio < 70%.

The PIFCOPD study received approval from the ethics review committees of Peking University First Hospital (2018 Number 31). Written informed consent was obtained from all participants.

### 2.2 Data collection

Data were collected from multiple sub-centers. Participants underwent anthropometric measurements, followed by pulmonary function testing. Anthropometric measurements, including height and weight, were performed by trained physicians according to standardized procedures across all centers. Body Mass Index (BMI) was calculated as weight in kilograms divided by the square of height in meters. Participants completed a set of standardized, self-administered questionnaires under the supervision of trained investigators. The standardized questionnaire used in this study is provided in Supplementary material S1. The questionnaires collected information on demographic and lifestyle characteristics, including sex, age, educational level, income, passive smoking exposure, smoking status, cumulative smoking exposure, biomass exposure, occupational exposure, household cooking habits, family history of COPD and asthma, geographic location and comorbidities. Passive smoking was defined as exposure to tobacco smoke by non-smokers in either the home or workplace. Smoking status was classified as current, former, or never smoker. Current smokers were individuals who had smoked ≥100 cigarettes in their lifetime and continued to smoke at the time of the survey (18). Former smokers had smoked ≥100 cigarettes in the past but had quit smoking prior to the survey. Never smokers were those who had smoked <100 cigarettes in their lifetime or had never smoked. Biomass exposure referred to the daily use of animal waste fuel or wood for heating or cooking. Occupational exposure refers to long-term contact with respiratory irritants such as dust, chemical fumes, and harmful gases in the workplace. Geographical location was categorized into northern and southern regions based on the Qinling Mountains-Huaihe River Line.

Pulmonary function was measured using the portable COPD-6 lung function instrument (COPD-6, Vitalograph Ltd., County Clare, Ireland). The test procedure has been described previously (17). The highest measurements of FEV_1_ and FEV_6_ from acceptable spirometry were included in the analysis. A bronchodilator test was performed if the FEV_1_:FEV_6_ ratio was less than 0.7. Quality control was performed by an expert panel based on the American Thoracic Society Committee on Proficiency Standards for Pulmonary Function Laboratories (ATS PFT Committee) 2017 standards (19).

### 2.3 Tea consumption

Data on tea consumption were collected through face-to-face interviews conducted by trained investigators. Participants were asked about their tea consumption habits, including whether they consumed tea, the types, frequency and duration of tea consumption. Tea types were classified based on their fermentation levels as follows: unfermented tea (predominantly green tea, including Longjing, Biluochun, and Pu-erh raw tea), partially fermented tea (including oolong tea, yellow tea, and white tea), fully fermented tea (predominantly black tea, including Congou black tea, Souchong black tea, and broken black tea), post-fermented tea (predominantly dark tea, including Pu-erh ripened tea, brick tea, and Tibetan tea), and reprocessed tea (specifically jasmine tea in this study). Tea consumption frequency was divided into three groups: 0, 1–6, and ≥ 7 times per week. One time of tea consumption was defined as using approximately 5–8 grams of dry tea leaves in a single brewing, which may yield multiple cups. The duration of tea consumption was similarly classified into three groups: 0, 1–9, and ≥ 10 years.

### 2.4 Statistical analyses

To compare characteristics between individuals with normal lung function and those with COPD, categorical variables were assessed using chi-square tests, continuous variables with normal distribution were analyzed with independent-sample t-tests, and non-normally distributed continuous or ordinal variables were analyzed using Mann–Whitney U tests. Descriptive statistics were presented as mean ± standard deviation for normally distributed data, and as median with interquartile range (IQR) for non-normally distributed data.

To examine the association between tea consumption and COPD, the analysis was conducted in two steps. The first step aimed to explore the association between tea consumption and the risk of COPD in the overall population. The second step further analyzed the interaction between tea consumption and smoking, with a stratified analysis based on smoking status. The purpose of the interaction analysis was to examine whether tea consumption and smoking have potential antagonistic or synergistic effects on COPD risk. In the stratified analysis, the aim was to assess the differential impact of tea consumption on COPD risk based on smoking status.

In the first step, we used multivariable logistic regression models to assess the association between tea consumption behaviors (type of tea, frequency of tea, and years of tea) and the risk of COPD in the overall population. The models were adjusted for known risk factors for COPD and other potential confounding factors, including sex, age, educational level, income, cumulative smoking exposure, biomass exposure, occupational exposure, household cooking habits, family history of asthma, family history of COPD, and geographic location. In the second step, we conducted interaction and stratified analyses based on smoking status, classifying participants as current/former smokers and never smokers. Multivariable logistic regression models were used to assess both interaction effects and stratified analyses. The models were adjusted for the same set of covariates as in the first step, excluding cumulative smoking exposure. Additionally, given that some participants consumed multiple types of tea, when analyzing the association between a specific type of tea and COPD risk, other types of tea were included as covariates to minimize potential confounding effects. Results of multivariable logistic regression models were presented as odds ratios (ORs) and 95% confidence intervals (CIs). All analyses were conducted using R version 4.4.2, with *p* value < 0.05 considered statistically significant.

## 3 Results

### 3.1 Demographic characteristics

The PIFCOPD study initially included 10,385 participants. After excluding participants with poor-quality spirometry data (*n* = 1,424), a total of 7,252 individuals were included in our study. Among them were participants with normal lung function (*n* = 6,855) and those diagnosed with COPD (*n* = 397). The overall characteristics and risk factors of the two groups are summarized in Table 1. Significant differences between the groups were found in sex, age, educational level, smoking status, cumulative smoking exposure, household cooking habits, family history of asthma, geographic region and hypertension. Compared with normal lung function group, the COPD group had more males (51.6% vs. 33.1%), an older median age (65 years vs. 61 years), and fewer participants with higher education (high school: 16.9% vs. 23.8%; college: 8.8% vs. 13.4%). Smoking was more prevalent in the COPD group (26.2% vs. 16.6%), with more pack-years and a greater family history of asthma (5.5% vs. 2.7%). Geographic region was also a distinguishing factor, with more participants with COPD living in southern regions (19.1% vs. 8.4%). The COPD group also had a higher rate of hypertension (34.2% vs. 24.2%). No significant differences were observed between the two groups in terms of BMI, income, biomass exposure, occupational exposure, family history of COPD or other comorbidities such as diabetes, dyslipidemia, heart disease, obstructive sleep apnea hypopnea syndrome, or cerebrovascular disease.

**Table 1:** Demographics, environmental exposures, and comorbidities by pulmonary function in the PIFCOPD population.

Variable	Normal lung function *N* = 6,855	COPD *N* = 397	*p* value
Sex			< 0.001
Male	2,270 (33.1)	205 (51.6)	
Female	4,585 (66.9)	192 (48.4)	
Age, years	61 (53, 66)	65 (57, 68)	< 0.001
BMI, kg/m^2^	24.5 (22.6, 26.8)	24.5 (22.5, 27.1)	0.918
Education level			< 0.001
No schooling or primary school	1,329 (19.4)	106 (26.7)	
Middle school	2,972 (43.4)	189 (47.6)	
High school	1,634 (23.8)	67 (16.9)	
College or higher	920 (13.4)	35 (8.8)	
Income, CNY			0.216
*<* 1,500	887 (12.9)	46 (11.6)	
1,500–4,499	5,059 (73.8)	291 (73.3)	
4,500–8,999	784 (11.5)	50 (12.6)	
≥ 9,000	125 (1.8)	10 (2.5)	
Passive smoking	667 (9.7)	41 (10.3)	0.762
Smoking type			*<*0.001
Never smoker	5,715 (83.4)	293 (73.8)	
Former or current smoker	1,140 (16.6)	104 (26.2)	
Cumulative smoking exposure, pack-years			*<* 0.001
0	5,715 (83.4)	293 (73.8)	
1–19	465 (6.8)	26 (6.4)	
≥ 20	675 (9.8)	78 (19.6)	
Biomass exposure	323 (4.7)	13 (3.3)	0.229
Occupational exposure	489 (7.1)	24 (6.1)	0.471
Household cooking	4,251 (62.0)	200 (50.4)	*<* 0.001
Geographic region			*<* 0.001
North	6,277 (91.6)	321 (80.9)	
South	578 (8.4)	76 (19.1)	
Family history of COPD	326 (4.8)	17 (4.3)	0.756
Family history of asthma	186 (2.7)	22 (5.5)	0.002
Comorbidities			
Hypertension	1,658 (24.2)	128 (32.2)	*<* 0.001
Diabetes	667 (9.7)	51 (12.8)	0.053
Hyperlipemia	239 (3.5)	11 (2.8)	0.536
Heart disease	264 (3.9)	19 (4.8)	0.423
OSAHS	66 (1.0)	3 (0.8)	0.883
Osteoporosis	74 (1.1)	2 (0.5)	0.400
Cerebrovascular disease	40 (0.6)	1 (0.3)	0.608

Data are presented median (IQR) for continuous variables and *n* (%) for categorical variables.

IQR, interquartile range. BMI, body mass index. OSAHS, obstructive sleep apnea hypopnea syndrome.

### 3.2 Tea consumption condition

**Figure 1:**
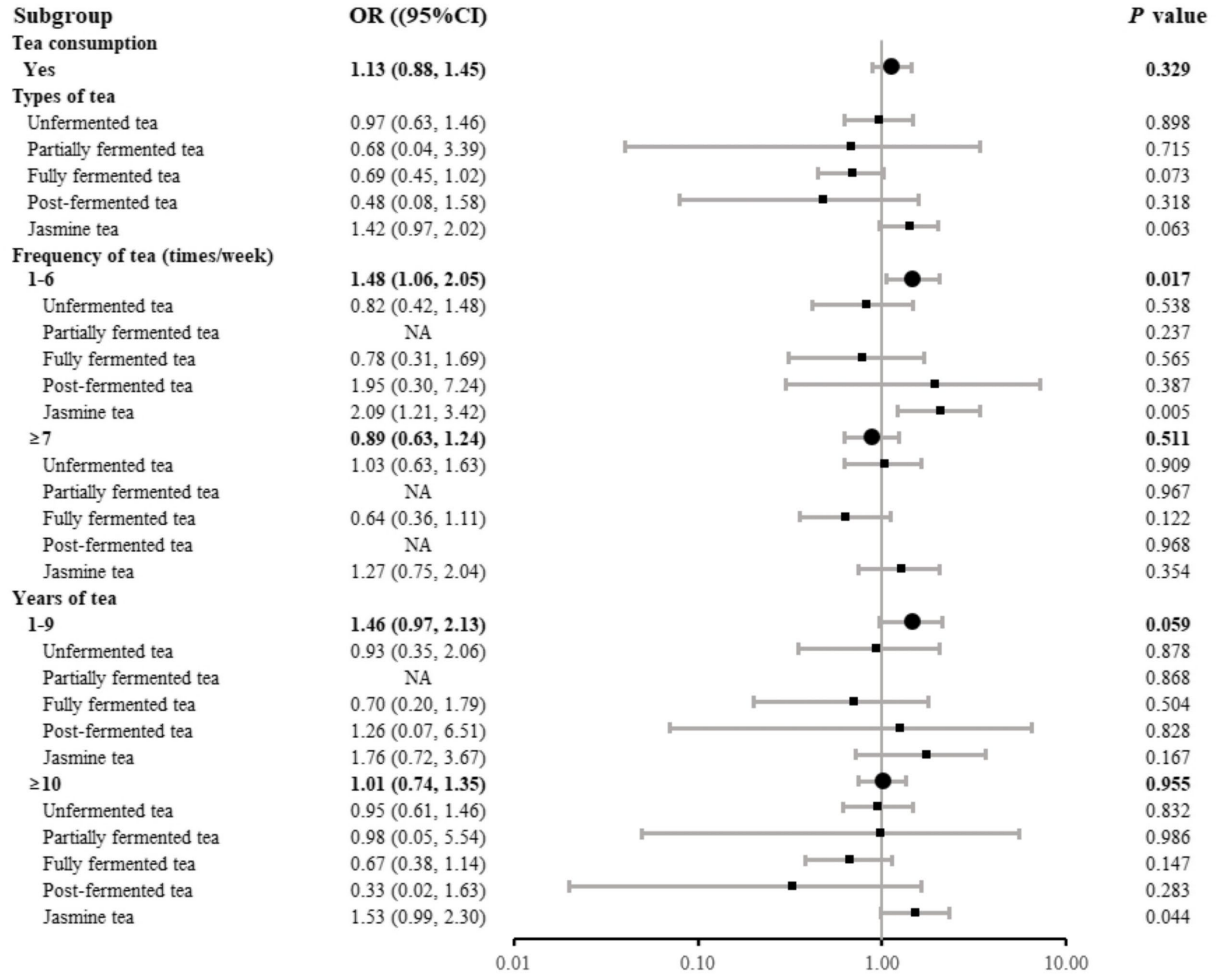
Adjusted odds ratios for COPD risk associated with tea consumption. Adjustment variables: sex, age, education level, income, pack-years of smoking, biomass exposure, occupational exposure, household cooking, family history of asthma, family history of COPD, and geographical location. In the analysis of the association between a specific type of tea and COPD risk, other types of tea are included as covariates. NA indicates that odds ratios could not be reliably estimated due to small sample size in the subgroup.

Tea consumption patterns, detailed in Table 2, showed no significant differences in total tea consumption or type of tea (unfermented tea, partially fermented tea, fully fermented tea or post-fermented tea) between groups, except for jasmine tea, where a significant difference was observed. The COPD group had more jasmine tea consumers (10.1% vs. 7.2%), with a higher frequency of consumption at 1–6 times per week (4.8% vs. 2.5%) and a longer duration of consumption (≥ 10 years: 8.3% vs. 5.9%). The relationship between tea consumption and the risk of COPD is illustrated in Figure 1. Adjusted logistic regression showed that drinking jasmine tea 1–6 times per week significantly increased the odds of COPD compared to non-tea consumers (OR 1.83, 95% CI: 1.06, 3.01, *p* = 0.022). No significant associations were observed between COPD risk and other types of tea.

**Table 2:** Tea consumption condition in individuals with normal lung function and COPD.

Variable	Normal lung function *N* = 6,855	COPD *N* = 397	*p* value
Tea consumption			0.102
Yes	1,430 (20.9)	97 (24.4)	
No	5,425 (79.1)	300 (75.6)	
Types of tea			
Unfermented tea	844 (12.3)	46 (11.6)	0.726
Partially fermented tea	25 (0.4)	1 (0.3)	*>* 0.999
Fully fermented tea	614 (9.0)	28 (7.1)	0.227
Post-fermented tea	70 (1.0)	2 (0.5)	0.453
Jasmine tea	493 (7.2)	40 (10.1)	0.041
Unfermented tea consumption			
Frequency of unfermented tea (times/week)			0.702
0	6,011 (87.7)	351 (88.4)	
1–6	265 (3.9)	12 (3.0)	
≥ 7	579 (8.4)	34 (8.6)	
Years of unfermented tea			0.696
0	6,011 (87.7)	351 (88.4)	
1–9	147 (2.1)	6 (1.5)	
≥ 10	697 (10.2)	40 (10.1)	
Partially fermented tea consumption			
Frequency of partially fermented tea (times/week)			0.713
0	6,830 (99.6)	396 (99.7)	
1–6	3 (0.1)	1 (0.3)	
≥ 7	22 (0.3)	0 (0)	
Years of partially fermented tea			0.715
0	6,830 (99.6)	396 (99.7)	
1–9	4 (0.1)	1 (0.3)	
≥ 10	21 (0.3)	0 (0)	
Fully fermented tea consumption			
Frequency of fully fermented tea (times/week)			0.197
0	6,241 (91.0)	369 (92.9)	
1–6	164 (2.4)	7 (1.8)	
≥ 7	450 (6.6)	21 (5.3)	
Years of fully fermented tea			0.198
0	6,241 (91.0)	369 (92.9)	
1–9	102 (1.5)	4 (1.0)	
≥ 10	512 (7.5)	24 (6.1)	
Post-fermented tea consumption			
Frequency of post-fermented tea (times/week)			0.309
0	6,785 (99.0)	395 (99.5)	
1–6	24 (0.3)	2 (0.5)	
≥ 7	46 (0.7)	0 (0)	
Years of post-fermented tea			0.311
0	6,785 (99.0)	395 (99.4)	
1–9	17 (0.2)	1 (0.3)	
≥ 10	53 (0.8)	1 (0.3)	
Jasmine tea consumption			
Frequency of jasmine tea (times/week)			0.039
0	6,362 (92.8)	357 (89.9)	
1–6	168 (2.5)	19 (4.8)	
≥ 7	325 (4.7)	21 (5.3)	
Years of jasmine tea			0.032
0	6,362 (92.8)	357 (89.9)	
1–9	89 (1.3)	7 (1.8)	
≥ 10	404 (5.9)	33 (8.3)	

Data are presented median (IQR) for continuous variables and *n* (%) for categorical variables.

Significant regional differences in tea consumption were observed. Northern participants showed higher overall tea intake, particularly unfermented and jasmine teas, with greater frequency and duration than southern participants, indicating distinct geographic tea-drinking patterns. Detailed tea consumption patterns are presented in Supplementary Table 1. Among participants with COPD, baseline demographic characteristics and tea consumption patterns were further compared between smokers and non-smokers. These descriptive data are summarized in Supplementary Table 2. Smokers showed a significantly higher prevalence of jasmine tea consumption than non-smokers.

### 3.3 Interaction and stratified analyses

**Figure 2:**
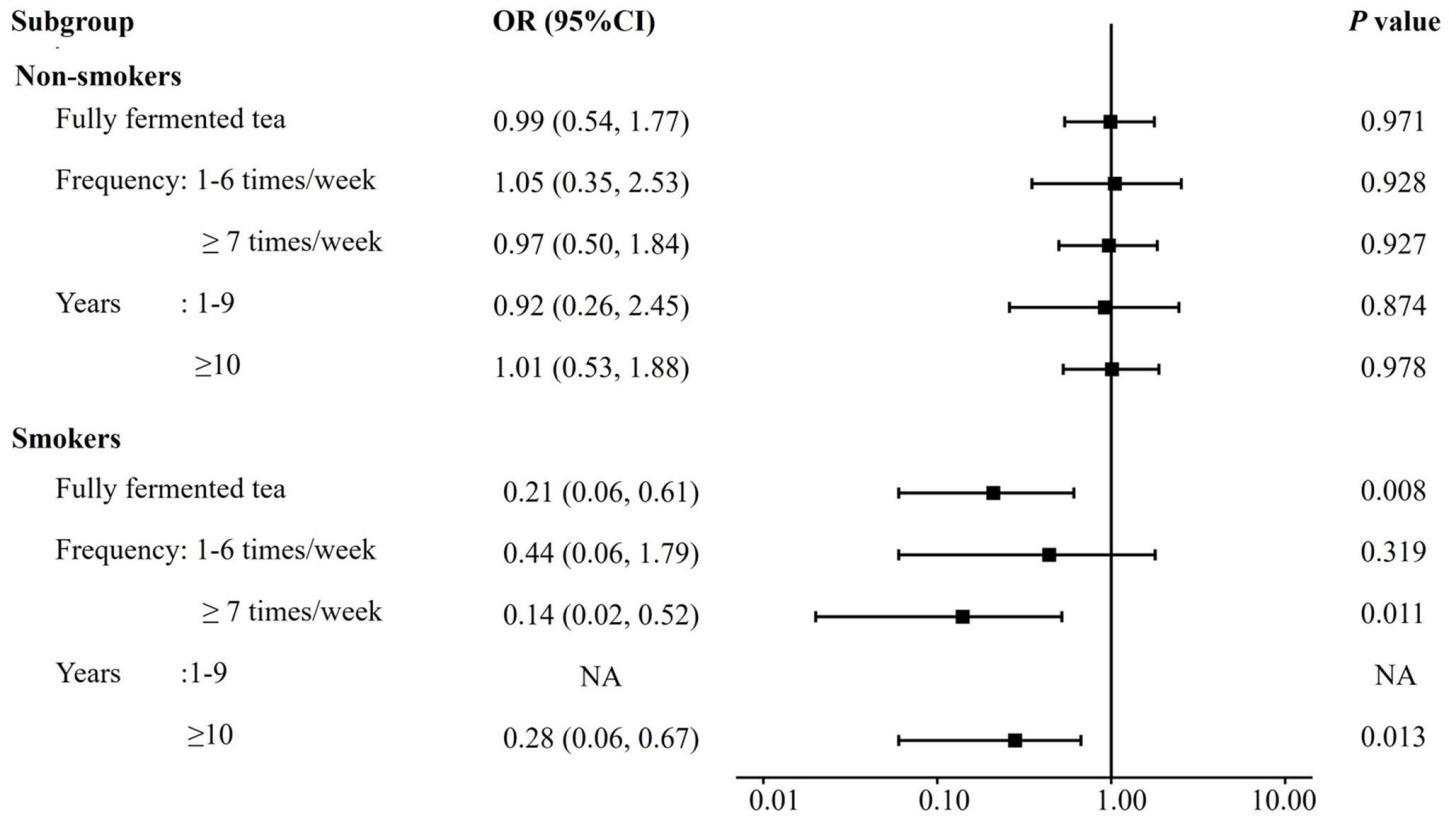
Odds ratios for COPD risk associated with fully fermented tea consumption, stratified by smoking status. Adjustment variables: sex, age, education level, income, biomass exposure, occupational exposure, household cooking, family history of asthma, family history of COPD, geographical location, unfermented tea, partially fermented tea, post-fermented tea and jasmine tea. NA indicates that odds ratios could not be reliably estimated due to small sample size in the subgroup.

The interaction between fully fermented tea and smoking status was presented in Table 3. Smoking significantly increased the risk of COPD prevalence (OR 1.69, 95% CI: 1.23, 2.33, *p* = 0.001), while fully fermented tea alone had no significant effect. However, fully fermented tea appeared to reduce the adverse effect of smoking on COPD risk (OR 0.20, 95% CI: 0.07, 0.62, *p* = 0.005), particularly among those who consumed it ≥ 7 times per week (OR 0.14, 95% CI: 0.03, 0.61, *p* = 0.010) or for ≥ 10 years (OR 0.25, 95% CI: 0.08, 0.78, *p* = 0.017). Further stratified analysis, shown in Figure 2, indicated that fully fermented tea did not affect COPD risk in non-smokers, but offered a protective effect in smokers (OR 0.21, 95% CI: 0.06, 0.61, *p* = 0.008), especially among those who consumed ≥ 7 times per week (OR 0.14, 95% CI: 0.02, 0.52, *p* = 0.011) or for ≥ 10 years (OR 0.28, 95% CI: 0.06, 0.67, *p* = 0.013).

**Table 3:** Interaction analysis between fully fermented tea and smoking on the risk of COPD.

Variable	Never-smoker		Smoker		Tea*smoking interaction	
	OR (95%CI)	*p* value	OR (95%CI)	*p* value	OR (95%CI)	*p* value
Fully fermented tea consumption						
No	1 (ref)		1.69 (1.23, 2.33)	0.001		
Yes	1.03 (0.60, 1.78)	0.901	0.35 (0.12, 1.04)	0.059	0.20 (0.07, 0.62)	0.005
Frequency of fully fermented tea(times/week)						
0	1 (ref)		1.69 (1.23, 2.33)	0.001		
1–6	1.11 (0.43, 2.88)	0.832	0.73 (0.16, 3.26)	0.679	0.39 (0.07, 2.16)	0.279
≥ 7	1.01 (0.56, 1.85)	0.963	0.23 (0.05, 1.00)	0.050	0.14 (0.03, 0.61)	0.010
Years of fully fermented tea						
0	1 (ref)		1.66 (1.20, 2.30)	0.002		
1–9	1.05 (0.36, 3.08)	0.925	NA	0.964	NA	0.962
≥ 10	1.03 (0.58, 1.85)	0.913	0.39 (0.14, 1.10)	0.075	0.25 (0.08, 0.78)	0.017

Adjustment variables: sex, age, education level, income, biomass exposure, occupational exposure, household cooking, family history of asthma, family history of COPD, geographical location, unfermented tea, partially fermented tea, post-fermented tea and jasmine tea. NA indicates that odds ratios could not be reliably estimated due to small sample size in the subgroup.

The interaction between unfermented tea consumption and smoking status was presented in Supplementary Table 3. Smoking significantly increased the risk of COPD prevalence (OR 1.66, 95% CI: 1.20, 2.30, *p* = 0.002). Unfermented tea consumption (including both frequency and duration) alone did not significantly affect the risk of COPD prevalence, but it interacted with smoking to reduce this risk (OR 0.48, 95% CI: 0.23, 0.99, *p* = 0.047), especially when consumed ≥ 7 times per week (OR 0.32, 95% CI: 0.13, 0.79, *p* = 0.014). However, this interaction does not fully offset the increased risk of smoking. Stratified analyses showed no significant association between unfermented tea and COPD risk in smokers or non-smokers (Supplementary Table 4).

**Figure 3:**
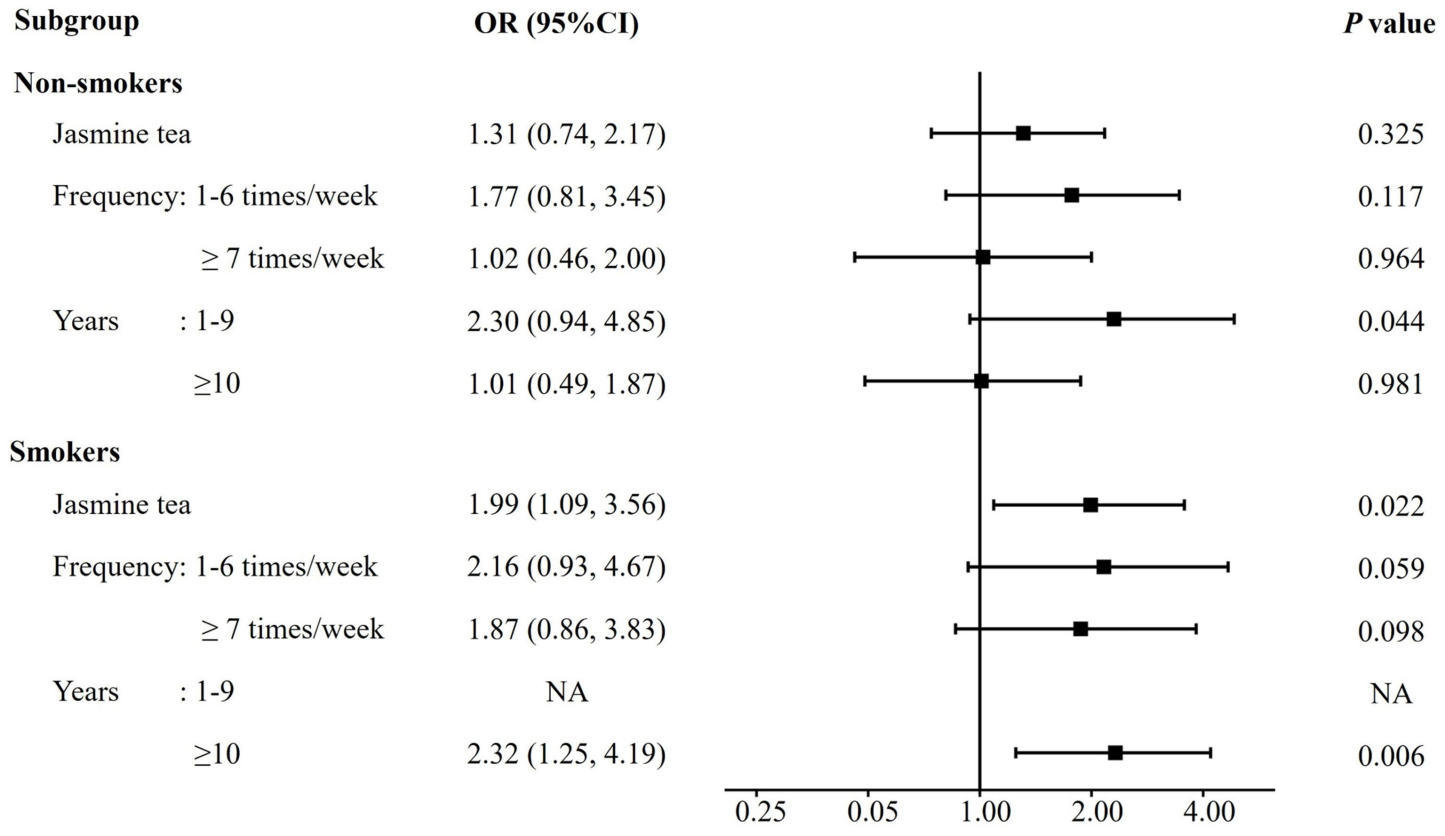
Odds ratios for COPD risk associated with jasmine tea consumption, stratified by smoking status. Adjustment variables: sex, age, education level, income, biomass exposure, occupational exposure, household cooking, family history of asthma, family history of COPD, geographical location, unfermented tea, partially fermented tea, fully fermented tea, and post-fermented tea. NA indicates that odds ratios could not be reliably estimated due to small sample size in the subgroup.

The interaction analysis between jasmine tea consumption and smoking status was shown in Supplementary Table 5. The main effect of jasmine tea on COPD was only significant for individuals with a consumption duration of 1–9 years (OR 2.44, 95% CI: 1.09, 5.50, *p* = 0.030). There was no significant interaction between jasmine tea and smoking. Stratified analysis, presented in Figure 3, showed no association between jasmine tea and COPD risk in non-smokers, but it was a risk factor for smokers (OR 1.99, 95% CI: 1.09, 3.56, *p* = 0.022), especially for those who had consumed it for ≥ 10 years (OR 2.32, 95% CI: 1.25, 4.19, *p* = 0.006). Partially fermented tea and post-fermented tea were not analyzed due to limited data. Considering the unequal distribution of participants across geographic regions, additional analyses restricted to the northern population were performed, and the results were broadly consistent with those observed in the overall population (Supplementary Tables 6–11).

## 4 Discussion

There were three main findings from this study. Firstly, an interaction between tea consumption and smoking status was observed in relation to prevalence of COPD, with this interaction depending on the specific type of tea consumed. Secondly, habitual and prolonged consumption of fully fermented tea was associated with a reduced risk of COPD among smokers, whereas prolonged consumption of jasmine tea was potentially associated with an increased risk. Thirdly, tea consumption alone was not associated with COPD risk in the general population, and no protective effect was observed among non-smokers.

Our study suggests that tea consumption may help counteract the risk of COPD among smokers, and this potential benefit appears to depend on the type of tea consumed. Smoking is a well-established risk factor for COPD, as it generates substantial amounts of ROS, which induce oxidative stress and chronic inflammation, thereby promoting airway remodeling and progressive decline in lung function (10, 20, 21). Tea, particularly due to its high polyphenol content, has been proposed to exert protective effects. However, most previous research on the interaction between tea consumption and smoking has focused on cancer. Studies have shown that regular drinking green or oolong tea may reduce the risk of lung, colorectal, oral or bladder cancer in smokers (22–25). However, evidence on this effect of tea on smoking-related chronic respiratory diseases is limited, although some laboratory studies suggest potential benefits. An analysis based on the Korean National Health and Nutrition Examination Survey (KNHANES) showed a negative trend between green tea intake and COPD risk among smokers, but the association did not reach statistical significance and detailed stratification by tea type or consumption pattern was lacking (26). In comparison, our study is the first to systematically examine the associations of different tea types, frequency, and duration of consumption with COPD risk using nationwide multicenter data, and to further investigate their interaction with smoking status.

Fully fermented tea showed a protective effect only in smokers, especially with regular and long-term consumption. Fully fermented tea, primarily black tea, undergoes extensive oxidation during processing, resulting in the conversion of most catechins to theaflavins and thearubigins (27). Theaflavins exhibit potent antioxidant properties, including scavenging free radicals, inhibiting lipid peroxidation, and upregulating antioxidant enzymes (28). Notably, their rate constants for superoxide scavenging exceed those of epigallocatechin-3-gallate (EGCG) (29). In addition, theaflavins exert anti-inflammatory effects through inhibition of the nuclear factor kappa-light-chain-enhancer of activated B cells (NF-κB) signaling pathway (30). Thearubigins further enhance antioxidant defense by activating the nuclear factor erythroid 2-related factor 2 (Nrf2)-mediated pathway and inducing phase II detoxifying enzymes in lung tissue (31). These mechanisms are supported by animal studies showing that fully fermented tea can attenuate cigarette smoke-induced oxidative stress, reduce alveolar damage, limit inflammatory cell infiltration and suppress apoptosis (32, 33). Together, these findings provide biological plausibility for the observed protective association between fully fermented tea and lower COPD risk among smokers.

In contrast, jasmine tea consumption (1–6 times per week) was associated with a higher risk of COPD among smokers. Jasmine tea, made by repeatedly scenting green tea with fresh jasmine (*Jasminum sambac*) flowers, has limited research on its biological effects. Some studies suggest that jasmine tea may have anti-inflammatory properties by downregulating tumor necrosis factor alpha (TNF-*α*) and NFκB, and its water-soluble polysaccharides have demonstrated antioxidant and free radical-scavenging effects (34, 35). The adverse effect of jasmine tea in our study may be due to impurities from the scenting process that may increase inflammation or oxidative stress. Additionally, the protective effects of jasmine tea’s bioactive components might diminish or become harmful under the high oxidative stress of smoking. These findings highlight the need for more research on long-term safety of reprocessed teas like jasmine tea, especially in vulnerable populations such as smokers.

Previous studies from Korea and Singapore have suggested that drinking green tea at least 2–3 times a day may reduce the risk of COPD in the general population (15, 16). However, our study found no significant association between unfermented tea consumption and COPD risk overall. There was a possible interaction between unfermented tea and smoking, but no protective effect was seen in smokers. Unfermented tea, primarily represented by green tea, is rich in polyphenols such as EGCG, both *in vitro* and *vivo* studies have shown that EGCG effectively attenuates oxidative stress and inflammation induced by cigarette smoke (36–38). The discrepancy between our findings and previous study may be due to the overwhelming oxidative burden of smoking, which may exceed the protective threshold of unfermented tea.

This study found that among smokers, regular (≥7 times per week) and long-term (≥10 years) consumption of fully fermented tea may help reduce the risk of COPD. Although smoking cessation is the cornerstone of COPD prevention, the 2018 China Adult Tobacco Survey reported a high smoking prevalence of 26.6% in China, with over 300 million smokers and a cessation rate of only 20.1%. In this context, from a clinical and public health perspective, fully fermented tea consumption may serve as a low-cost and culturally acceptable adjunctive lifestyle factor for COPD risk modification among smokers. Further large-scale epidemiological studies, particularly well-designed prospective cohort studies and randomized controlled trials targeting high-risk smokers with standardized assessment of tea type, dose, and duration, are warranted to validate the protective effects of tea consumption against COPD.

This study has several limitations. Firstly, due to its cross-sectional design, causal relationships between tea consumption and COPD risk cannot be established. Secondly, data on tea consumption were self-reported, which may be subject to recall bias. Additionally, the number of participants consuming certain tea types was limited, restricting further stratified or interaction analyses. Thirdly, the substantially larger number of participants with normal lung function compared with those with COPD reflects the population-based design of the study in the general community. The higher proportion of female participants may be related to higher participation and response rates among women in community-based health surveys, whereas the unequal geographic distribution, with fewer participants from southern China, was due to the limited number of participating centers in that region. These factors may affect the generalizability of the findings. However, sex and geographic region were adjusted for in the multivariable analyses, and regional differences in tea consumption patterns were further explored. Future studies should recruit larger and more diverse populations to facilitate more comprehensive subgroup analyses.

## 5 Conclusion

This study highlights the complex interplay between different types of tea and smoking in modulating COPD risk. Among smokers, fully fermented tea-particularly when consumed at a frequency of ≥ 7 times per week and sustained for ≥ 10 years-was associated with a significant protective effect against COPD. Jasmine tea consumption for ≥ 10 years was associated with an increased risk of COPD. These findings offer new evidence on the tea-COPD relationship and provide a theoretical foundation for developing targeted preventive strategies in populations at elevated risk for COPD due to smoking.

## Data Availability

The raw data supporting the conclusions of this article will be made available by the authors, without undue reservation.
