# Supplementary material for: Heterogeneous impact of tea consumption on COPD risk in smokers: insights from the PIFCOPD study
Source: Front Med (Lausanne). 2026 Mar 18;13:1776347. doi: 10.3389/fmed.2026.1776347 (PMC13039063; doi:10.3389/fmed.2026.1776347)
Supplement: Supplementary file 1 [file Table_1.docx]

Supplementary Material

# Supplementary Material S1

**Questionnaire**

**General Information**

- **Date of birth:** ____ / ____ / ____ (Year / Month / Day)
- **Age:** ____ years
- **Sex:** ☐ Male ☐ Female
- **Home address:** __________________________________________
  *(to the level of residential community)*
- **Work unit (if not retired):** _____________________________
- **Ethnicity:**
  ☐ Han
  ☐ Ethnic minority (please specify: ____________)
- **Marital status:**
  ☐ Never married
  ☐ Married
  ☐ Divorced
  ☐ Widowed
- **Education level:**
  ☐ Primary school or below
  ☐ Middle school
  ☐ High school / Technical secondary school
  ☐ Junior college
  ☐ Bachelor’s degree or above
- **Occupation (current or previous):**
  ☐ Government employee
  ☐ Farmer
  ☐ Teacher
  ☐ Military personnel
  ☐ Factory worker
  ☐ Physician
  ☐ Other occupations not otherwise classified
- **Average monthly household income per capita (RMB):**
  ☐ < 1,500
  ☐ 1,500–4,499
  ☐ 4,500–8,999
  ☐ 9,000–19,999
  ☐ ≥ 20,000

**Past Medical History and Medication Use**

- **History of the following diseases (check all that apply):**
  ☐ Hypertension
  ☐ Diabetes mellitus
  ☐ Dyslipidemia
  ☐ Cardiovascular disease
  ☐ Obesity
  ☐ Obstructive sleep apnea–hypopnea syndrome
  ☐ Osteoporosis
  ☐ Other (please specify: ____________)

**Risk Factors**

**1. Tobacco Smoke Exposure**

☐ Never smoked

☐ Current smoker

- - Duration: ____ years
  - Average number of cigarettes per day: ____
  - Other tobacco products: ☐ Water pipe ☐ Hand-rolled tobacco ☐ Cigar

☐ Former smoker

- - Quit duration: ____ years / months
  - Total smoking duration: ____ years
  - Average number of cigarettes per day: ____
  - Other tobacco products: ☐ Water pipe ☐ Hand-rolled tobacco ☐ Cigar

☐ Passive smoking exposure

- - Duration: ____ years
    *(Definition: non-smokers exposed to tobacco smoke for ≥15 minutes on at least 1 day per week)*

**2. Biomass Fuel Smoke Exposure**

*(e.g., use of firewood, charcoal, crop residues, or animal dung for cooking or heating)*

- Exposure duration: ____ years
- Time since cessation of exposure: ____ years

**3. Household Cooking Exposure**

- Duration of household cooking: ____ years
- Kitchen ventilation equipment:
  ☐ Yes ☐ No

**4. Occupational Environmental Exposure**

- Exposure duration: ____ years
- Time since cessation of exposure: ____ years
- **Type of occupational exposure (multiple choices allowed):**
  ☐ Crop farming (rice, cotton, etc.)
  ☐ Crop farming (vegetables, fruits, etc.)
  ☐ Mining or quarrying
  ☐ Foundry work
  ☐ Plastics, resins, paints, or related industries
  ☐ Carpentry
  ☐ Flour processing
  ☐ Textile industry
  ☐ Radiation exposure
  ☐ Poultry breeding (pigeons, chickens, ducks, etc.)
  ☐ Livestock farming
  ☐ Other (please specify: _____________________)

**Tea Consumption**

☐ Yes

- - Duration of tea consumption: ____ years
  - Main type of tea:
    ☐ Black tea
    ☐ Green tea
    ☐ Dark tea
    ☐ Oolong tea
    ☐ Flower tea
  - Average weekly frequency (per brewing): ____ times

**Family History**

- **Family history of COPD or emphysema:**
  ☐ Yes ☐ No ☐ Unknown
- **Family history of asthma:**
  ☐ Yes ☐ No ☐ Unknown

# Supplementary Tables

Supplementary Table 1 Regional differences in tea consumption between northern and southern China

|  | North  N=6598 | South  N=654 | *p* value |
| --- | --- | --- | --- |
| Tea consumption |  |  | < 0.001 |
| Yes | 1449 (22.0) | 78 (11.9) |  |
| No | 5149 (78.0) | 576 (88.1) |  |
| Types of tea |  |  |  |
| Unfermented tea | 827 (12.5) | 63 (9.6) | 0.036 |
| Partially fermented tea | 25 (0.4) | 1 (0.2) | 0.562 |
| Fully fermented tea | 596 (7.0) | 46 (9.0) | 0.100 |
| Post-fermented tea | 70 (1.1) | 2 (0.3) | 0.099 |
| Jasmine tea | 531 (8.1) | 2 (0.3) | < 0.001 |
| Unfermented tea consumption |  |  |  |
| Frequency of unfermented tea (times/week) |  |  | 0.029 |
| 0 | 5771 (87.4) | 591 (90.3) |  |
| 1-6 | 255 (3.9) | 22 (3.4) |  |
| ≥ 7 | 572 (8.7) | 41 (6.3) |  |
| Years of unfermented tea |  |  | 0.020 |
| 0 | 5771 (87.4) | 591 (90.4) |  |
| 1-9 | 131 (2.0) | 22 (3.4) |  |
| ≥ 10 | 696 (10.6) | 41 (6.2) |  |
| Partially fermented tea consumption |  |  |  |
| Frequency of partially fermented tea (times/week) |  |  | 0.355 |
| 0 | 6573 (99.6) | 653 (99.8) |  |
| 1-6 | 3 (0.1) | 0 (0) |  |
| ≥ 7 | 22 (0.3) | 1 (0.2) |  |
| Years of partially fermented tea |  |  | 0.357 |
| 0 | 6573 (99.6) | 653 (99.8) |  |
| 1-9 | 4 (0.1) | 1 (0.2) |  |
| ≥ 10 | 21 (0.3) | 0 (0) |  |
| Fully fermented tea consumption |  |  |  |
| Frequency of fully fermented tea (times/week) |  |  | 0.087 |
| 0 | 6002 (91.0) | 608 (93.0) |  |
| 1-6 | 159 (2.4) | 12 (1.8) |  |
| ≥ 7 | 437 (6.6) | 34 (5.2) |  |
| Years of fully fermented tea |  |  | 0.054 |
| 0 | 6002 (91.0) | 608 (92.9) |  |
| 1-9 | 82 (1.3) | 24 (3.7) |  |
| ≥ 10 | 514 (7.8) | 22 (3.4) |  |
| Post-fermented tea consumption |  |  |  |
| Frequency of post-fermented tea (times/week) |  |  | 0.064 |
| 0 | 6528 (98.9) | 652 (99.7) |  |
| 1-6 | 26 (0.4) | 0 (0) |  |
| ≥ 7 | 44 (0.7) | 2 (0.3) |  |
| Years of post-fermented tea |  |  | 0.063 |
| 0 | 6528 (98.9) | 652 (99.7) |  |
| 1-9 | 17 (0.3) | 1 (0.2) |  |
| ≥ 10 | 53 (0.8) | 1 (0.2) |  |
| Jasmine tea consumption |  |  |  |
| Frequency of jasmine tea (times/week) |  |  | < 0.001 |
| 0 | 6067 (92.0) | 652 (99.7) |  |
| 1-6 | 185 (2.8) | 2 (0.3) |  |
| ≥ 7 | 346 (5.2) | 0 (0) |  |
| Years of jasmine tea |  |  | < 0.001 |
| 0 | 6067 (92.0) | 652 (99.7) |  |
| 1-9 | 94 (1.4) | 2 (0.3) |  |
| ≥ 10 | 437 (6.6) | 0 (0) |  |

Data are presented median (IQR) for continuous variables and n (%) for categorical variables.

Supplementary Table 2 Demographic characteristics and tea consumption patterns among smokers and non-smokers with COPD

|  | COPD non-smokers  N=293 | COPD smokers  N=104 | *p* value |
| --- | --- | --- | --- |
| Sex |  |  | < 0.001 |
| Male | 103 (35.2)) | 102 (98.1) |  |
| Female | 190 (64.8%) | 2 (1.9) |  |
| Age, years | 65 (57, 68) | 66 (58, 68) | 0.516 |
| BMI, kg/m2 | 24.8 (22.7, 27.1) | 23.8 (22.3, 26.1) | 0.010 |
| Education level |  |  | 0.776 |
| No schooling or primary school | 83 (28.3) | 23 (22.1) |  |
| Middle school | 128 (43.7) | 61 (58.7) |  |
| High school | 54 (18.4) | 13 (12.5) |  |
| College or higher | 28 (9.6) | 7 (6.7) |  |
| Income, CNY |  |  | 0.729 |
| *<* 1500 | 33 (11.3) | 13 (12.5) |  |
| 1500-4499 | 218 (74.4) | 73 (70.2) |  |
| 4500-8999 | 36 (12.3) | 14 (13.5) |  |
| ≥ 9000 | 6 (2.1) | 4 (3.9) |  |
| Geographic region |  |  | 0.201 |
| North | 232 (79.2) | 89 (85.6) |  |
| South | 61 (20.8) | 15 (14.4) |  |
| Tea consumption |  |  | 0.412 |
| Yes | 68 (23.2) | 29 (27.9) |  |
| No | 225 (76.8) | 75 (72.1) |  |
| Types of tea |  |  |  |
| Unfermented tea | 33 (11.3) | 13 (12.5) | 0.873 |
| Partially fermented tea | 1 (0.3) | 0 (0.0) | >0.999 |
| Fully fermented tea | 24 (8.2) | 4 (3.9) | 0.206 |
| Post-fermented tea | 1 (0.3) | 1 (1.0) | >0.999 |
| Jasmine tea | 18 (6.1) | 22 (21.2) | < 0.001 |
| Unfermented tea consumption |  |  |  |
| Frequency of unfermented tea (times/week) |  |  | 0.819 |
| 0 | 260 (88.7) | 91 (87.5) |  |
| 1-6 | 6 (2.1) | 6 (5.8) |  |
| ≥ 7 | 27 (9.2) | 7 (6.7) |  |
| Years of unfermented tea |  |  | 0.715 |
| 0 | 260 (88.7) | 91 (87.5) |  |
| 1-9 | 5 (1.7) | 1 (1.0) |  |
| ≥ 10 | 28 (9.6) | 12 (11.5) |  |
| Partially fermented tea consumption |  |  |  |
| Frequency of partially fermented tea (times/week) |  |  | 0.555 |
| 0 | 292 (99.7) | 104 (100) |  |
| 1-6 | 1 (0.3) | 0 (0.0) |  |
| ≥ 7 | 0 (0.0) | 0 (0.0) |  |
| Years of partially fermented tea |  |  | 0.555 |
| 0 | 292 (99.7) | 104 (100) |  |
| 1-9 | 0 (0.0) | 0 (0.0) |  |
| ≥ 10 | 1 (0.3) | 0 (0.0) |  |
| Fully fermented tea consumption |  |  |  |
| Frequency of fully fermented tea (times/week) |  |  | 0.129 |
| 0 | 269 (91.8) | 100 (96.2) |  |
| 1-6 | 5 (1.7) | 2 (1.9) |  |
| ≥ 7 | 19 (6.5) | 2 (1.9) |  |
| Years of fully fermented tea |  |  | 0.143 |
| 0 | 269 (91.8) | 100 (96.2) |  |
| 1-9 | 4 (1.4) | 0 (0.0) |  |
| ≥ 10 | 20 (6.8) | 4 (3.9) |  |
| Post-fermented tea consumption |  |  |  |
| Frequency of post-fermented tea (times/week) |  |  | 0.446 |
| 0 | 292 (99.7) | 103 (99.0) |  |
| 1-6 | 1 (0.3) | 1 (1.0) |  |
| ≥ 7 | 0 (0.0) | 0 (0.0) |  |
| Years of post-fermented tea |  |  | 0.443 |
| 0 | 292 (99.7) | 103 (99.0) |  |
| 1-9 | 1 (0.3) | 0 (0.0) |  |
| ≥ 10 | 0 (0.0) | 1 (1.0) |  |
| Jasmine tea consumption |  |  |  |
| Frequency of jasmine tea (times/week) |  |  | < 0.001 |
| 0 | 275 (93.9) | 82 (78.8) |  |
| 1-6 | 9 (3.1) | 10 (9.6) |  |
| ≥ 7 | 9 (3.1) | 12 (11.5) |  |
| Years of jasmine tea |  |  | <0.001 |
| 0 | 275 (93.9) | 82 (78.8) |  |
| 1-9 | 7 (2.4) | 0 (0.0) |  |
| ≥ 10 | 11 (3.8) | 22 (21.2) |  |

Data are presented median (IQR) for continuous variables and n (%) for categorical variables.

IQR, interquartile range. BMI, body mass index.

Supplementary Table 3 Interaction analysis between unfermented tea and smoking on the risk of COPD

|  | Never-smoker | |  | Smoker | |  | Tea*smoking interaction | |
| --- | --- | --- | --- | --- | --- | --- | --- | --- |
|  | OR (95%CI) | *p* value |  | OR (95%CI) | *p* value |  | OR (95%CI) | *p* value |
| Unfermented tea consumption | | | | | | | | |
| No | 1 (ref) |  |  | 1.66 (1.20, 2.30) | 0.002 |  |  |  |
| Yes | 1.25 (0.78, 1.98) | 0.353 |  | 1.00 (0.51, 1.94) | 0.047 |  | 0.48 (0.23, 0.99) | 0.047 |
| Frequency of unfermented tea(times/week) | | | | | | | | |
| 0 | 1 (ref) |  |  | 1.66 (1.20, 2.30) | 0.002 |  |  |  |
| 1-6 | 0.76 (0.33, 1.79) | 0.534 |  | 1.49 (0.59, 3.81) | 0.399 |  | 1.17 (0.35, 3.95) | 0.797 |
| ≥ 7 | 1.51 (0.90, 2.56) | 0.122 |  | 0.83 (0.35, 1.96) | 0.672 |  | 0.32 (0.13, 0.79) | 0.014 |
| Years of unfermented tea | | | | | | | | |
| 0 | 1 (ref) |  |  | 1.66 (1.20, 2.30) | 0.002 |  |  |  |
| 1-9 | 1.12 (0.43, 2.90) | 0.811 |  | 0.69 (0.09, 5.51) | 0.730 |  | 0.35 (0.04, 3.29) | 0.361 |
| ≥ 10 | 1.28 (0.78, 2.10) | 0.332 |  | 1.07 (0.53, 2.14) | 0.850 |  | 0.49 (0.23, 1.05) | 0.067 |

Adjustment variables: sex, age, education level, income, biomass exposure, occupational exposure, household cooking, family history of asthma, family history of COPD, geographical location, partially fermented tea, fully fermented tea, post-fermented tea and jasmine tea.

Supplementary Table 4 Odds ratios for COPD risk and unfermented tea consumption, stratified by smoking status

|  | Never-smoker |  |  | Smoker |  | |  |
| --- | --- | --- | --- | --- | --- | --- | --- |
|  | OR (95%CI) | *p* value |  | OR (95%CI) | *p* value | |  |
| Unfermented tea consumption | | | | | |  |  |
| No | 1 (ref) |  |  | 1 (ref) |  | | |
| Yes | 1.03 (0.60, 1.71) | 0.898 |  | 0.95 (0.45, 1.87) | 0.894 | |  |
| Frequency of unfermented tea(times/week) | | | | | |  |  |
| 0 | 1 (ref) |  |  | 1 (ref) |  | |  |
| 1-6 | 0.71 (0.27, 1.56) | 0.442 |  | 1.15 (0.41, 2.76) | 0.777 | |  |
| ≥ 7 | 1.23 (0.67, 2.17) | 0.490 |  | 0.82 (0.31, 1.92) | 0.669 | |  |
| Years of unfermented tea | | | | | |  |  |
| 0 | 1 (ref) |  |  | 1 (ref) |  | |  |
| 1-9 | 0.93 (0.31, 2.25) | 0.883 |  | 0.73 (0.04, 4.19) | 0.773 | |  |
| ≥ 10 | 1.06 (0.60, 1.80) | 0.837 |  | 0.98 (0.45, 1.96) | 0.955 | |  |

Adjustment variables: sex, age, education level, income, biomass exposure, occupational exposure, household cooking, family history of asthma, family history of COPD, geographical location, partially fermented tea, fully fermented tea, post-fermented tea and jasmine tea.

Supplementary Table 5 Interaction analysis between jasmine tea and smoking on the risk of COPD

|  | Never-smoker | |  | Smoker | |  | Tea*smoking interaction | |
| --- | --- | --- | --- | --- | --- | --- | --- | --- |
|  | OR (95%CI) | *p* value |  | OR (95%CI) | *p* value |  | OR (95%CI) | *p* value |
| Jasmine tea consumption | | | | | | | | |
| No | 1 (ref) |  |  | 1.46 (1.05, 2.03) | 0.024 |  |  |  |
| Yes | 1.48 (0.88, 2.49) | 0.139 |  | 2.45 (1.45, 4.15) | *<*0.001 |  | 1.14 (0.55, 2.32) | 0.728 |
| Frequency of jasmine tea(times/week) | | | | | | | | |
| 0 | 1 (ref) |  |  | 1.46 (1.05, 2.03) | 0.024 |  |  |  |
| 1-6 | 1.88 (0.92-3.85) | 0.081 |  | 3.54 (1.68, 7.46) | *<*0.001 |  | 1.25 (0.45, 3.47) | 0.666 |
| ≥ 7 | 1.20 (0.59-2.44) | 0.614 |  | 1.98 (1.01, 3.87) | 0.046 |  | 1.11 (0.43, 2.88) | 0.823 |
| Years of jasmine tea | | | | | | | | |
| 0 | 1 (ref) |  |  | 1.46 (1.05, 2.03) | 0.024 |  |  |  |
| 1-9 | 2.44 (1.09, 5.50) | 0.030 |  | NA | 0.972 |  | NA | 0.953 |
| ≥ 10 | 1.18 (0.62, 2.24) | 0.622 |  | 2.67 (1.57, 4.54) | *<*0.001 |  | 1.57 (0.70, 3.54) | 0.275 |

Adjustment variables: sex, age, education level, income, biomass exposure, occupational exposure, household cooking, family history of asthma, family history of COPD, geographical location, unfermented tea, partially fermented tea, fully fermented tea and post-fermented tea.

NA indicates that odds ratios could not be reliably estimated due to small sample size in the subgroup.

Supplementary Table 6 Interaction analysis between fully fermented tea and smoking on the risk of COPD in the northern population

|  | Never-smoker | |  | Smoker | |  | Tea*smoking interaction | |
| --- | --- | --- | --- | --- | --- | --- | --- | --- |
|  | OR (95%CI) | *p* value |  | OR (95%CI) | *p* value |  | OR (95%CI) | *p* value |
| Fully fermented tea consumption | | | | | | | | |
| No | 1 (ref) |  |  | 1.50 (1.10, 2.05) | 0.011 |  |  |  |
| Yes | 0.94 (0.58, 1.53) | 0.805 |  | 0.41 (0.15, 1.14) | 0.088 |  | 0.29 (0.09, 0.91) | 0.033 |
| Frequency of fully fermented tea(times/week) | | | | | | | | |
| 0 | 1 (ref) |  |  | 1.50 (1.09, 2.05) | 0.011 |  |  |  |
| 1-6 | 1.13 (0.40, 3.15) | 0.817 |  | 0.55 (0.10, 3.17) | 0.504 |  | 0.37 (0.06, 2.16) | 0.267 |
| ≥ 7 | 0.90 (0.52, 1.55) | 0.701 |  | 0.34 (0.07, 1.54) | 0.161 |  | 0.23 (0.05, 0.95) | 0.047 |
| Years of fully fermented tea | | | | | | | | |
| 0 | 1 (ref) |  |  | 1.50 (1.10, 2.05) | 0.011 |  |  |  |
| 1-9 | 0.53 (0.07, 3.89) | 0.532 |  | NA | 0.971 |  | NA | 0.970 |
| ≥ 10 | 0.98 (0.59, 1.63) | 0.951 |  | 0.45 (0.15, 1.39) | 0.165 |  | 0.30 (0.10, 0.95) | 0.041 |

Adjustment variables: sex, age, education level, income, biomass exposure, occupational exposure, household cooking, family history of asthma, family history of COPD, geographical location, unfermented tea, partially fermented tea, post-fermented tea and jasmine tea. NA indicates that odds ratios could not be reliably estimated due to small sample size in the subgroup.

Supplementary Table 7 Odds ratios for COPD risk and fully fermented tea consumption in the northern population, stratified by smoking status

|  | Never-smoker |  |  | Smoker |  |
| --- | --- | --- | --- | --- | --- |
|  | OR (95%CI) | *p* value |  | OR (95%CI) | *p* value |
| Fully fermented tea consumption | | | | | |
| No | 1 (ref) |  |  | 1 (ref) |  |
| Yes | 0.92 (0.57, 1.51) | 0.750 |  | 0.33 (0.11, 0.94) | 0.037 |
| Frequency of fully fermented tea(times/week) | | | | | |
| 0 | 1 (ref) |  |  | 1 (ref) |  |
| 1-6 | 1.02 (0.36, 2.85) | 0.974 |  | 0.51 (0.11, 2.22) | 0.366 |
| ≥ 7 | 0.90 (0.52, 1.56) | 0.708 |  | 0.24 (0.06, 0.99) | 0.045 |
| Years of fully fermented tea | | | | | |
| 0 | 1 (ref) |  |  | 1 (ref) |  |
| 1-9 | 0.45 (0.06, 3.31) | 0.432 |  | NA | 0.987 |
| ≥ 10 | 0.98 (0.59, 1.63) | 0.945 |  | 0.35 (0.12, 0.98) | 0.047 |

Adjustment variables: sex, age, education level, income, biomass exposure, occupational exposure, household cooking, family history of asthma, family history of COPD, geographical location, unfermented tea, partially fermented tea, post-fermented tea and jasmine tea. NA indicates that odds ratios could not be reliably estimated due to small sample size in the subgroup.

Supplementary Table 8 Interaction analysis between jasmine tea and smoking on the risk of COPD in the northern population

|  | Never-smoker | |  | Smoker | |  | Tea*smoking interaction | |
| --- | --- | --- | --- | --- | --- | --- | --- | --- |
|  | OR (95%CI) | *p* value |  | OR (95%CI) | *p* value |  | OR (95%CI) | *p* value |
| Jasmine tea consumption | | | | | | | | |
| No | 1 (ref) |  |  | 1.30 (0.93, 1.81) | 0.123 |  |  |  |
| Yes | 1.35 (0.81, 2.25) | 0.247 |  | 1.82 (1.09, 3.04) | 0.021 |  | 1.04 (0.50, 2.15) | 0.916 |
| Frequency of jasmine tea(times/week) | | | | | | | | |
| 0 | 1 (ref) |  |  | 1.30 (0.93, 1.81) | 0.123 |  |  |  |
| 1-6 | 1.89 (0.93, 3.84) | 0.081 |  | 1.37 (0.51, 3.70) | 0.533 |  | 1.06 (0.38, 2.96) | 0.916 |
| ≥ 7 | 1.05 (0.52, 2.10) | 0.901 |  | 1.40 (0.56, 3.50) | 0.478 |  | 1.08 (0.41, 2.79) | 0.882 |
| Years of jasmine tea | | | | | | | | |
| 0 | 1 (ref) |  |  | 1.30 (0.93, 1.81) | 0.123 |  |  |  |
| 1-9 | 2.48 (1.11, 5.56) | 0.027 |  | NA | 0.970 |  | NA | 0.969 |
| ≥ 10 | 1.04 (0.55, 1.97) | 0.899 |  | 1.91 (0.88, 4.16) | 0.101 |  | 1.48 (0.65, 3.35) | 0.348 |

Adjustment variables: sex, age, education level, income, biomass exposure, occupational exposure, household cooking, family history of asthma, family history of COPD, geographical location, unfermented tea, partially fermented tea, fully fermented tea and post-fermented tea. NA indicates that odds ratios could not be reliably estimated due to small sample size in the subgroup.

Supplementary Table 9 Odds ratios for COPD risk and jasmine tea consumption in the northern population, stratified by smoking status

|  | Never-smoker |  |  | Smoker |  |
| --- | --- | --- | --- | --- | --- |
|  | OR (95%CI) | *p* value |  | OR (95%CI) | *p* value |
| Jasmine tea consumption | | | | | |
| No | 1 (ref) |  |  | 1 (ref) |  |
| Yes | 1.28 (0.77, 2.13) | 0.348 |  | 1.54 (0.87, 2.72) | 0.138 |
| Frequency of jasmine tea(times/week) | | | | | |
| 0 | 1 (ref) |  |  | 1 (ref) |  |
| 1-6 | 1.77 (0.87, 3.61) | 0.116 |  | 1.88 (0.85, 4.16) | 0.117 |
| ≥ 7 | 1.00 (0.50, 2.01) | 0.994 |  | 1.34 (0.66, 2.71) | 0.423 |
| Years of jasmine tea | | | | | |
| 0 | 1 (ref) |  |  | 1 (ref) |  |
| 1-9 | 2.32 (1.04, 5.19) | 0.040 |  | NA | 0.981 |
| ≥ 10 | 0.98 (0.52, 1.86) | 0.960 |  | 1.73 (1.07, 3.09) | 0.042 |

Adjustment variables: sex, age, education level, income, biomass exposure, occupational exposure, household cooking, family history of asthma, family history of COPD, geographical location, unfermented tea, partially fermented tea, fully fermented tea and post-fermented tea. NA indicates that odds ratios could not be reliably estimated due to small sample size in the subgroup.

Supplementary Table 10 Interaction analysis between unfermented tea and smoking on the risk of COPD in the northern population

|  | Never-smoker | |  | Smoker | |  | Tea*smoking interaction | |
| --- | --- | --- | --- | --- | --- | --- | --- | --- |
|  | OR (95%CI) | *p* value |  | OR (95%CI) | *p* value |  | OR (95%CI) | *p* value |
| Unfermented tea consumption | | | | | | | | |
| No | 1 (ref) |  |  | 1.48 (1.07, 2.04) | 0.017 |  |  |  |
| Yes | 1.06 (0.70, 1.61) | 0.767 |  | 0.91 (0.48, 1.72) | 0.763 |  | 0.58 (0.27, 1.23) | 0.154 |
| Frequency of unfermented tea(times/week) | | | | | | | | |
| 0 | 1 (ref) |  |  | 1.48 (1.07, 2.04) | 0.017 |  |  |  |
| 1-6 | 0.94 (0.40, 2.18) | 0.882 |  | 1.07 (0.31, 3.71) | 0.912 |  | 0.73 (0.20, 2.57) | 0.619 |
| ≥ 7 | 1.11 (0.70, 1.76) | 0.671 |  | 0.76 (0.31, 1.88) | 0.557 |  | 0.52 (0.20, 1.31) | 0.164 |
| Years of unfermented tea | | | | | | | | |
| 0 | 1 (ref) |  |  | 1.48 (1.07, 2.04) | 0.017 |  |  |  |
| 1-9 | 0.94 (0.29, 3.04) | 0.922 |  | 0.84 (0.08, 8.74) | 0.885 |  | 0.57 (0.05, 5.96) | 0.638 |
| ≥ 10 | 1.08 (0.70, 1.67) | 0.724 |  | 0.85 (0.40, 1.80) | 0.669 |  | 0.57 (0.26, 1.27) | 0.169 |

Adjustment variables: sex, age, education level, income, biomass exposure, occupational exposure, household cooking, family history of asthma, family history of COPD, geographical location, partially fermented tea, fully fermented tea, post-fermented tea and jasmine tea. NA indicates that odds ratios could not be reliably estimated due to small sample size in the subgroup.

Supplementary Table 11 Odds ratios for COPD risk and unfermented tea consumption in the northern population, stratified by smoking status

|  | Never-smoker |  |  | Smoker |  |
| --- | --- | --- | --- | --- | --- |
|  | OR (95%CI) | *p* value |  | OR (95%CI) | *p* value |
| Unfermented tea consumption | | | | | |
| No | 1 (ref) |  |  | 1 (ref) |  |
| Yes | 1.04 (0.69, 1.58) | 0.837 |  | 0.77 (0.40, 1.51) | 0.453 |
| Frequency of unfermented tea(times/week) | | | | | |
| 0 | 1 (ref) |  |  | 1 (ref) |  |
| 1-6 | 0.88 (0.38, 2.05) | 0.768 |  | 0.81 (0.30, 2.16) | 0.666 |
| ≥ 7 | 1.10 (0.69, 1.75) | 0.684 |  | 0.75 (0.32, 1.75) | 0.508 |
| Years of unfermented tea | | | | | |
| 0 | 1 (ref) |  |  | 1 (ref) |  |
| 1-9 | 0.85 (0.26, 2.74) | 0.784 |  | 0.86 (0.11, 7.04) | 0.890 |
| ≥ 10 | 1.08 (0.69, 1.66) | 0.745 |  | 0.77 (0.38, 1.54) | 0.453 |

Adjustment variables: sex, age, education level, income, biomass exposure, occupational exposure, household cooking, family history of asthma, family history of COPD, geographical location, partially fermented tea, fully fermented tea, post-fermented tea and jasmine tea. NA indicates that odds ratios could not be reliably estimated due to small sample size in the subgroup.
